# Single-Level Posterior Lumbar Interbody Fusion for Scoliosis Associated With Postural Imbalance and Low Back Pain: A Case Report

**DOI:** 10.7759/cureus.113418

**Published:** 2026-07-26

**Authors:** Hiroto Koba, Toshio Doi

**Affiliations:** 1 Orthopedic Surgery I, Hiroshima Red Cross Hospital & Atomic-bomb Survivors Hospital, Hiroshima, JPN; 2 Orthopedic Surgery II, Hiroshima Red Cross Hospital & Atomic-bomb Survivors Hospital, Hiroshima, JPN

**Keywords:** antalgic scoliosis, coronal imbalance, degenerative lumbar scoliosis, posterior lumbar interbody fusion, radiculopathy

## Abstract

Elderly patients often experience a severely impaired quality of life due to chronic pain and truncal shift. While spinal deformities primarily caused by radiculopathy secondary to degenerative diseases can often be managed with focal intervention, they are frequently misdiagnosed as permanent structural issues. This radiculopathy-induced "antalgic scoliosis" presents a significant diagnostic challenge; identifying and treating the localized pain trigger may allow patients to avoid extensive reconstructive surgery. We report the case of a 66-year-old female with pre-existing degenerative scoliosis who presented with an acute, severe exacerbation of coronal imbalance. A diagnostic selective nerve root block provided transient but complete relief of both pain and truncal tilt, confirming the L4 nerve root as the primary antalgic driver of the coronal malalignment. A single-level posterior lumbar interbody fusion (PLIF) at L4/5 achieved immediate restoration of global coronal and sagittal alignment and significant improvement in radiographic parameters. Identifying focal triggers of antalgic posture via selective blocks is crucial; targeted single-level fusion can occasionally resolve global imbalance, sparing elderly patients from the significant morbidity associated with extensive reconstructive surgery.

## Introduction

Spinal scoliosis is characterized by frontal plane curvature, often presenting with shoulder imbalance and truncal deformity. Clinical management depends heavily on differentiating between functional and structural scoliosis. In the elderly, degenerative lumbar scoliosis (DLS) is common, and global sagittal or coronal imbalance often leads to recommendations for extensive thoracolumbar-pelvic fusion. However, radiculopathy can cause a reflexive truncal shift, referred to as "sciatic" or "painful" scoliosis, to alleviate nerve root compression. We present a case where a single-level posterior lumbar interbody fusion (PLIF) successfully treated a severe coronal imbalance by addressing a localized radicular trigger in a patient with underlying structural scoliosis.

## Case presentation

A 66-year-old female (height: 151 cm; weight: 45 kg) was referred to our department with a primary complaint of low back pain, tension in the left thigh, and a progressive rightward truncal tilt. Her symptoms progressively exacerbated over the preceding year, becoming refractory to conservative management, including pharmacotherapy and caudal epidural blocks. Her medical history included Sjögren's syndrome and lumbar spondylolytic spondylolisthesis.

Physical examination revealed a marked rightward truncal shift. Neurological examination revealed weakness in the left extensor hallucis longus (EHL) muscle, graded as 4/5 on the Manual Muscle Testing (MMT) scale [1]; however, no sensory deficits or pathological reflexes were elicited.

Imaging findings

**Figure 1:**
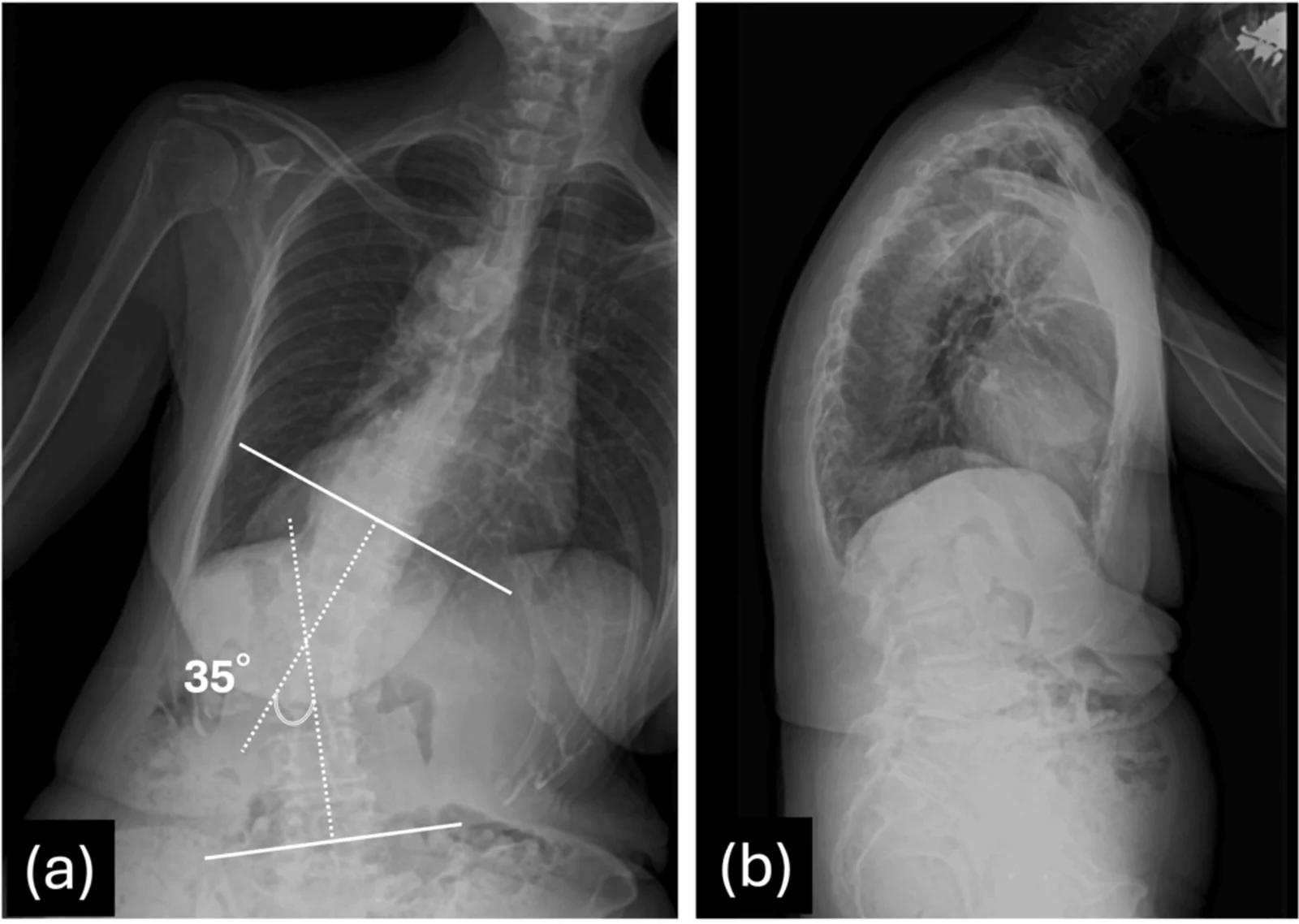
Initial radiographic assessment of the spine. (a) Standing whole-spine X-ray in the coronal view demonstrating degenerative lumbar scoliosis with a Cobb angle of 35 (T11-L4, apex at L2). A marked rightward truncal shift and global coronal imbalance are evident. (b) Sagittal view showing increased kyphosis and significant sagittal malalignment.

**Figure 2:**
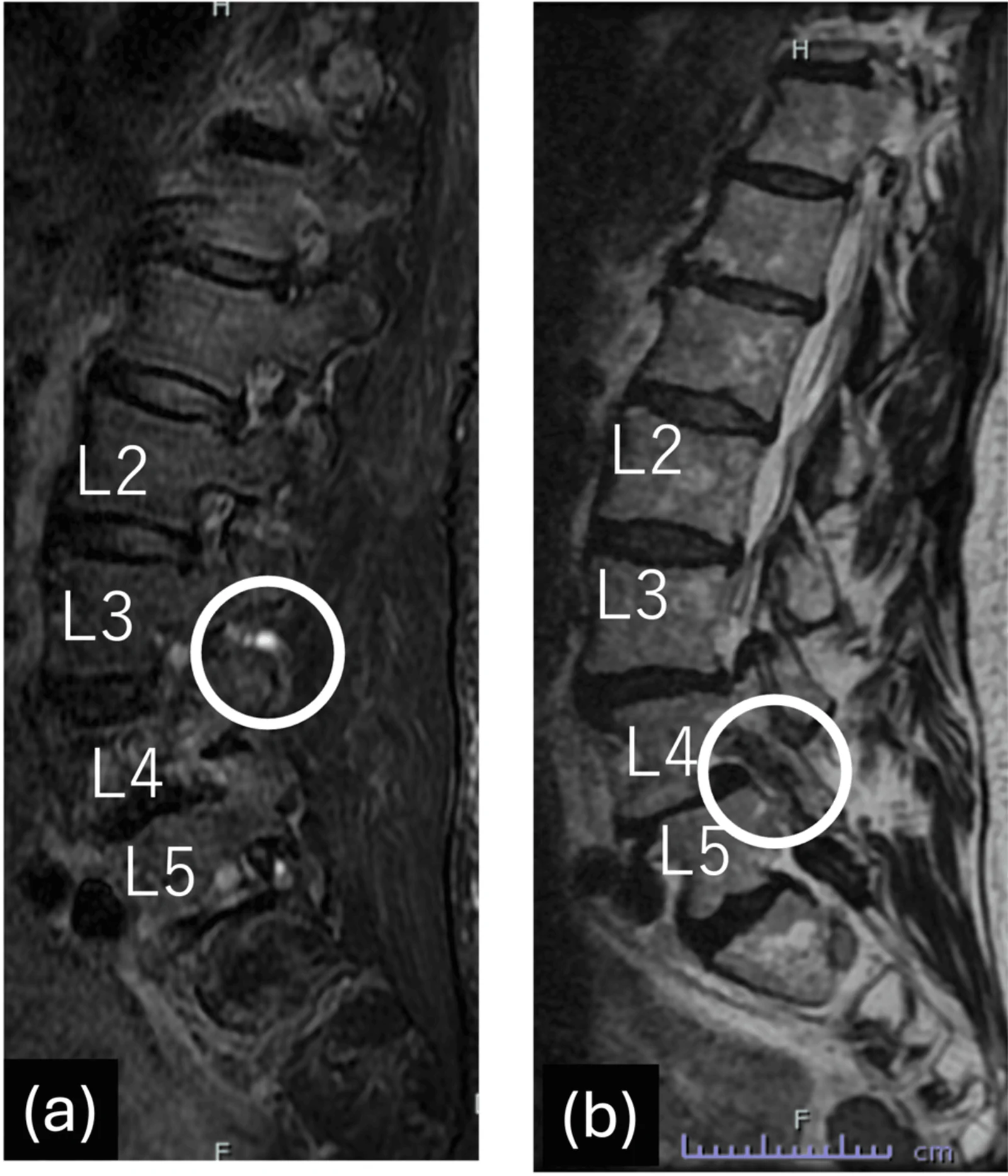
Preoperative MRI findings of the lumbar spine. Preoperative MRI showing (a) left L3/4 facet joint effusion, (b) flattening and posterior bulging of the L4/5 intervertebral disc, stenosis of the left L4/5 neural foramen.

**Figure 3:**
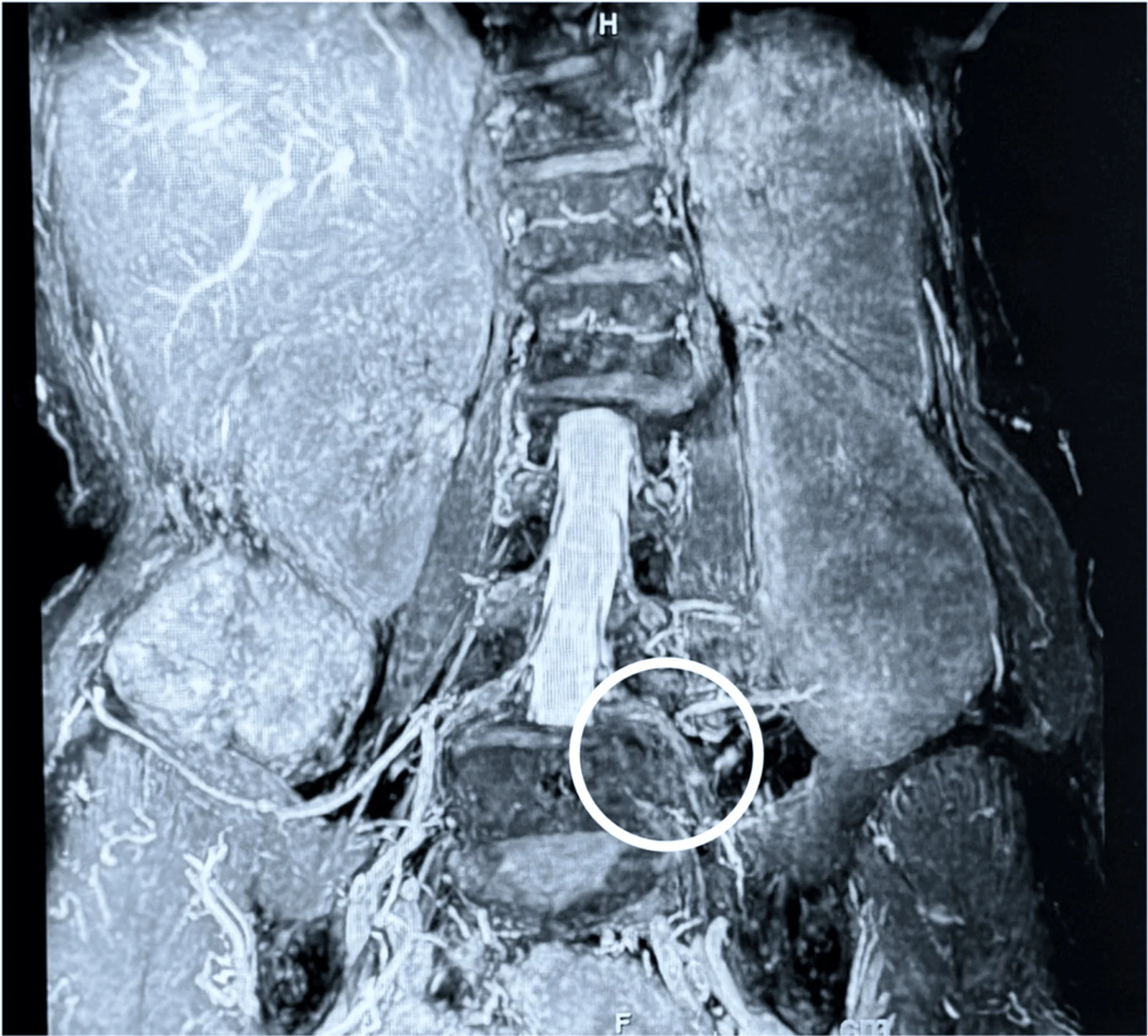
COSMIC FatSAT radial MRI findings. COSMIC FatSAT radial MRI showing horizontalization of the left L4 nerve root. COSMIC, coherent oscillatory state acquisition for the manipulation of imaging contrast; FatSAT, fat saturation.

Plain radiography demonstrated a left-convex degenerative scoliosis (from T11 to L4 with apex at L2) with a Cobb angle of 35° (Figure 1a) and increased kyphosis (Figure 1b). MRI demonstrated only mild central canal stenosis; however, significant findings included left L3/4 facet joint effusion (Figure 2a), flattening and posterior bulging of the L4/5 disc, and narrowing of the left L4/5 neural foramen (Figure 2b). Furthermore, COSMIC (coherent oscillatory state acquisition for the manipulation of imaging contrast) FatSAT (fat saturation) radial MRI demonstrated distinct kinking or horizontal displacement of the left L4 nerve root (Figure 3).

Clinical course

A diagnostic left L3/4 facet block failed to provide relief. However, a left L4 selective nerve root block resulted in the immediate disappearance of the low back pain and a visible improvement in the truncal tilt. Consequently, we diagnosed the patient with painful scoliosis (functional) superimposed on a pre-existing degenerative scoliosis (structural), primarily driven by left L4/5 foraminal stenosis.

**Figure 4:**
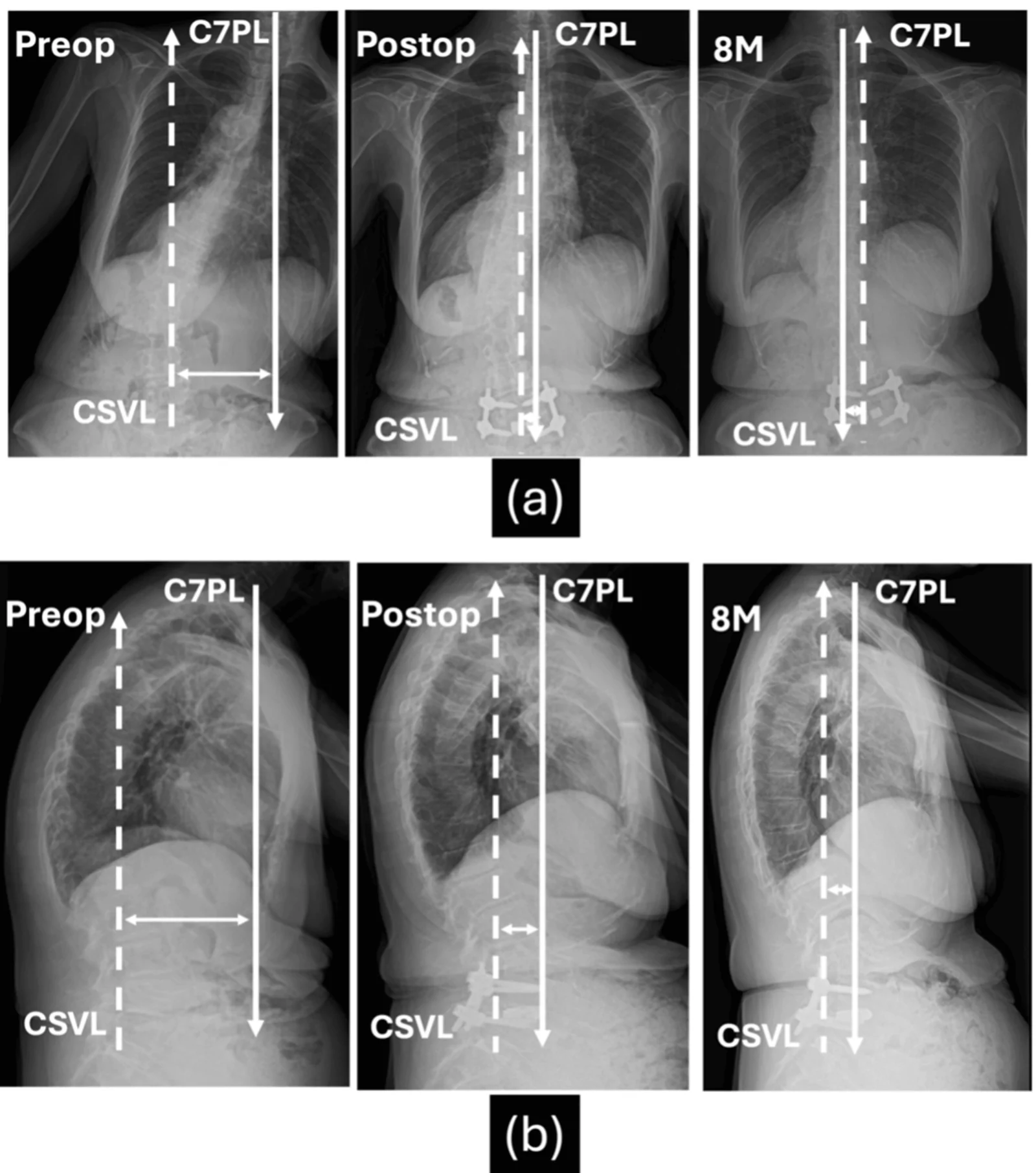
Postoperative radiographs. Postoperative standing radiographs showing (a) the medial migration of the C7 plumb line (C7PL) toward the midline and (b) the reduction of the C7PL shift on the lateral view.

**Figure 5:**
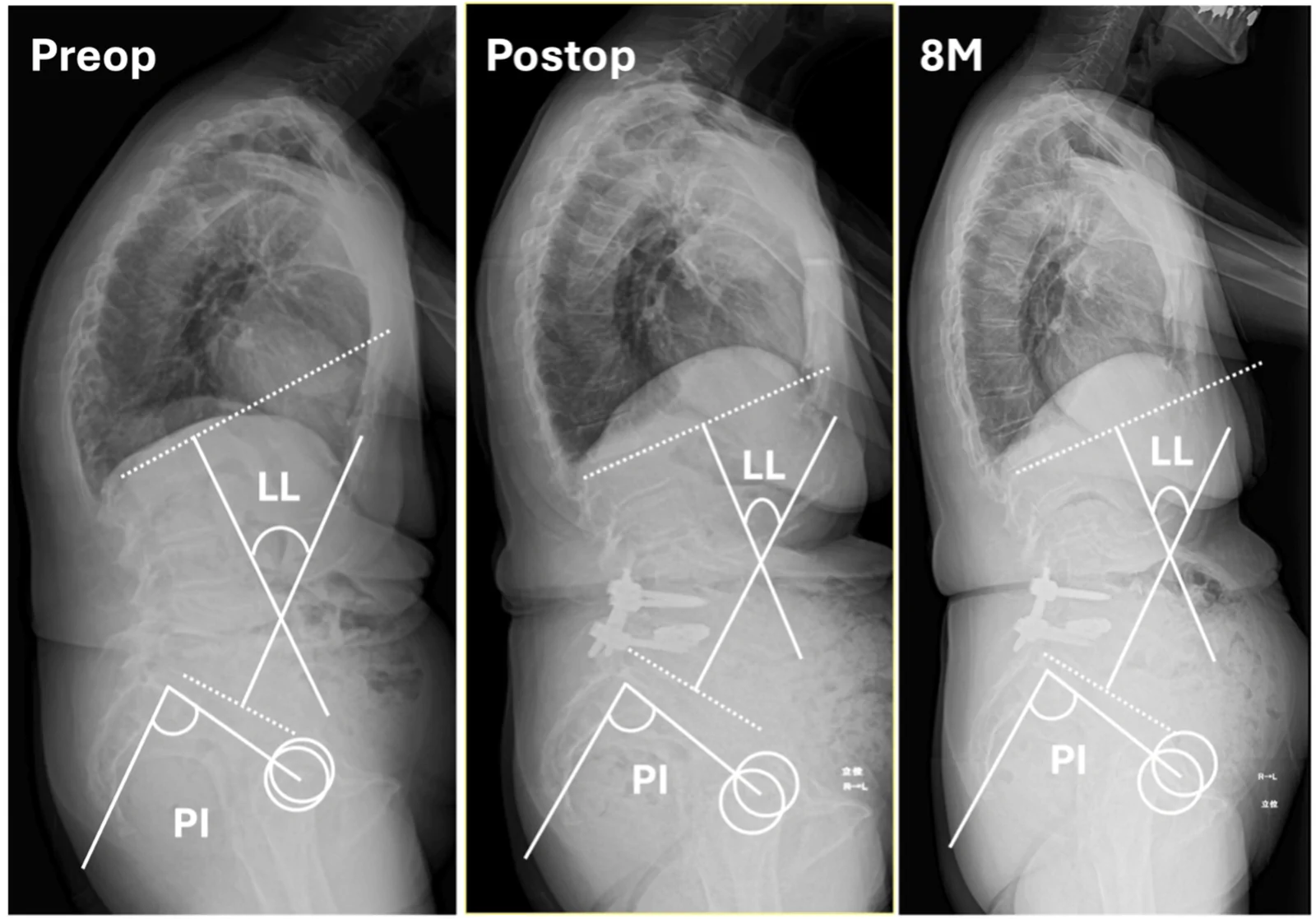
Postoperative standing radiograph. Postoperative standing lateral radiograph showing the reduction of the pelvic incidence-lumbar lordosis (PI-LL) mismatch.

The patient underwent a single-level PLIF at L4/5. Immediately following surgery, the radicular pain resolved and the truncal shift was corrected, allowing for independent ambulation. Postoperative whole-spine radiographs showed that the C7 plumb line had returned to the midline (Figure 4a), the C7PL sagittal shift had shortened (Figure 4b), and the PI-LL mismatch was reduced (Figure 5). At the eight-month follow-up, the patient maintained excellent activities of daily living (ADL) without recurrence of pain or deformity. Furthermore, radiographic parameters and standardized functional outcome measures demonstrated significant sustained improvements (Tables 1, 2).

**Table 1:** Radiographic parameters before and after surgery. Summary of spinopelvic parameters demonstrating immediate and sustained postoperative correction of C7PL-shift and PI-LL mismatch up to 8 months follow-up. C7PL: C7 plumb line; AP: anteroposterior; Lat: lateral; PI-LL: pelvic incidence minus lumbar lordosis.

Parameter	Preoperative	Postoperative	Eight months follow-up
C7PL-shift (AP) (mm)	105.84	10.48	-20.33
C7PL-shift (Lat) (mm)	103.7	63.01	30.23
PI-LL (°)	27	9	9

**Table 2:** Standardized functional outcome measures. Summary of clinical outcomes showing marked improvements in ODI percentage score, VAS for low back pain, and EQ-5D index value at 8 months follow-up. ODI, Oswestry Disability Index [2]; VAS, Visual Analogue Scale [3]; EQ-5D, EuroQol 5 Dimensions [4].

Parameter	Preoperative	Eight months follow-up
ODI percentage score	46.7	20
VAS for low back pain	8	1
EQ-5D index value	0.609	0.831

## Discussion

Degenerative scoliosis has become increasingly prevalent among the aging population, particularly among women over the age of 60 years [5]. In cases of degenerative scoliosis with spinal malalignment, existing literature highlights that long-segment stabilization from the thoracic spine to the ilium has been reported to improve postoperative functional outcomes [6]. However, such extensive surgeries carry high complication rates in elderly patients.

Painful scoliosis is characterized by a reflexive shift in axial posture triggered by pain, most commonly secondary to neurological irritation such as L4/5 lumbar disc herniation [7]. The scoliotic deformity is often reversible and resolves following the successful decompression or resolution of the radiculopathy [8].

In the present case, we hypothesized the complex spinal deformity to be a combination of pre-existing DLS (structural) manifesting in middle age and a superimposed painful scoliosis (functional) resulting from an antalgic adaptation to left L4 radiculopathy. Our treatment strategy prioritized a staged intervention. We initially performed focal decompression and stabilization directed at the symptomatic neural level to address the functional component of the deformity (the painful scoliosis). A secondary, more extensive corrective fusion from the thoracic spine to the pelvis, targeting the underlying degenerative scoliosis, was reserved only for persistent truncal imbalance.

Ultimately, single-level stabilization alone achieved comprehensive restoration of global spinal alignment, effectively obviating the necessity for a secondary, more invasive long-segment reconstruction. The prompt restoration of global alignment in this case suggests that the pre-existing coronal malalignment was largely an antalgic adaptation rather than a fixed structural deformity. By addressing the symptomatic L4 nerve root through single-level PLIF, we eliminated the antalgic trigger, thereby neutralizing the compensatory truncal shift and allowing the patient to regain a physiological global balance. In elderly patients presenting with truncal malalignment and concomitant degenerative scoliosis, it is imperative to discern the extent to which an evasive posture, driven by radicular pain, contributes to the overall deformity. A meticulous preoperative assessment of this functional component is paramount to avoiding over-surgery and minimizing surgical morbidity.

## Conclusions

We successfully treated a patient with degenerative scoliosis complicated by painful functional scoliosis. A single-level PLIF addressed the nerve root irritation, which in turn resolved the global truncal imbalance. In the management of elderly spinal deformity, identifying focal triggers of antalgic posture is essential to providing effective, minimally invasive surgical solutions.
